# Differential regulation of cellular functions by the C-termini of transmembrane 4 L six family proteins in 2- or 3-dimensional environment

**DOI:** 10.18632/oncotarget.14809

**Published:** 2017-01-25

**Authors:** Jin-Gyu Cheong, Dae-Geun Song, Haeng Eun Song, Fedor Berditchevski, Seo Hee Nam, Jae Woo Jung, Hye-Jin Kim, Ji Eon Kim, Somi Kim, Jihye Ryu, Chang Yun Cho, Kyung-Min Lee, Jung Weon Lee

**Affiliations:** ^1^ Department of Pharmacy, Research Institute of Pharmaceutical Sciences, College of Pharmacy, Seoul National University, Seoul 08826, Republic of Korea; ^2^ Systems Biotechnology Research Center, Korea Institute of Science and Technology (KIST), Gangneung-si, Gangwon-do 25451, Republic of Korea; ^3^ Institute of Cancer and Genomic Sciences, University of Birmingham, Edgbaston, Birmingham B15 2TT, UK; ^4^ Interdisciplinary Program in Genetic Engineering, Seoul National University, Seoul 08826, Republic of Korea

**Keywords:** 3D cell culture, migration, proliferation, spheroids, transmembrane 4 L six family

## Abstract

The transmembrane 4 L six family proteins TM4SF1, TM4SF4, and TM4SF5 share 40-50% overall sequence identity, but their C-terminus identity is limited. It may be likely that the C-termini of the members are important and unique for own regulatory functions. We thus examined how the TM4SF5 C-terminus affected cellular functions differentially from other family members. Using colon cancer cells expressing wildtype (WT), C-terminus-deleted, or chimeric mutants, diverse cellular functions were explored in 2-dimensional (2D) and 3-dimensional (3D) condition. The C-termini of the proteins were relatively comparable with respect to 2D cell proliferation, although each C-terminal-deletion mutant exhibited increased proliferation relative to the WT. Using chimeric constructs, we found that the TM4SF5 C-terminus was critical for regulating the diverse metastatic functions of TM4SF5, and could positively replace the C-termini of other family members. Replacement of the TM4SF1 or TM4SF4 C-terminus with that of TM4SF5 increased spheroids growth, transwell migration, and invasive dissemination from spheroids in 3D collagen gels. TM4SF5-mediated effects required its extracellular loop 2 linked to the C-terminus via the transmembrane domain 4, with causing c-Src activation. Altogether, the C-terminus of TM4SF5 appears to mediate pro-migratory roles, depending on a structural relay from the second extracellular loop to the C-terminus.

## INTRODUCTION

The plasma membranes sense extracellular environmental cues that trigger the intracellular signaling pathways to regulate cellular behaviors. The membrane is thought to contain a diverse array of protein “communities” that function in sensing extracellular cues. These protein communities include focal adhesions, lipid rafts, and tetraspanin-enriched microdomains (TERMs) [1]. Membrane proteins in these communities form complexes with other membrane proteins (or receptors) and/or cytosolic proteins to transduce intracellular signals upon extracellular stimulations [2]. The protein complexes also modulate the trafficking and/or stability of the components [3]. TERMs-mediated signaling activity is particularly important, as it regulates cell functions such as proliferation, migration, and gene expression [2].

The transmembrane 4 L six family, which is similar to the tetraspanins, includes TM4SF1 (L6, L6-Ag), TM4SF4 (IL-TMP), TM4SF5 (L6H), TM4SF18 (L6D), and TM4SF20 [4]. mRNA or protein levels of TM4SF1 are enhanced in liver, cervical, lung, colon, breast, prostate, and squamous cell cancers [5–7]. TM4SF1 in liver cancer cells promotes proliferation, invasion, and metastasis [7]. Furthermore, TM4SF1 in breast cancer cells couples the collagen I receptor tyrosine kinase DDR1 to the syntenin 2, a cortical adaptor, and further PKCα that promotes phosphorylation and activation of JAK2 and STAT3 for cancer stem cell traits and metastatic reactivation in the metastatic sites such as lung, bone, and brain [8]. Meanwhile, TM4SF4 is expressed in non-dividing hepatocytes and upregulated during liver injury possibly for cellular differentiation and regeneration [9–11], although it is highly enhanced in liver cancer [12]. Enhanced TM4SF4 level in lung carcinoma cells can lead to resistance to radiotherapy via IGF1-induced IGFR activation [13]. However, TM4SF4 in intestinal epithelium and liver is shown to be a negative regulator of cell proliferation [9, 11]. Thus, roles of TM4SF4 in cell proliferation are not clear.

These proteins have four transmembrane domains (TMs), two extracellular loops (EC1 for the short extracellular loop and EC2 for the long extracellular loop), an intracellular loop (ICL), and two cytosolic (N- and C-) termini. The EC2 of TM4SF5 can be *N*-glycosylated at N138 and N155, and its *N*-glycosylation status is important for the interaction with other receptors such as integrin α2 or CD44 [14, 15]. Meanwhile, the ICL or the C-terminus of TM4SF5 interacts with focal adhesion kinase (FAK) or c-Src to direct cellular migration or invasive protrusion, respectively [16, 17]. The cytosolic portion of TM4SF5 also interacts with the cytosolic tail of integrin α5 to induce signaling for vascular endothelial growth factor induction [18]. The TMs of TM4SF1 are involved in targeting the protein into late endocytic organelles, and interaction between the C-terminus of TM4SF1 and cytosolic syntenin-2 is critical for targeting TM4SF1 to the TERM [19]. However, the roles of the subdomains in the other transmembrane 4 L six family members remain unknown.

TM4SF1, TM4SF4, and TM4SF5 exhibit 40-50% overall sequence identity, which is thereby sufficient for them to share a common membrane topology. However, the sequences of the C-termini differ substantially, which may differentially play roles in regulating the cellular functions via activation of intracellular signaling pathways in manners unique in each member and/or differential among the members [4, 20]. In other words, it may be likely that the C-termini of the members are important and unique for own regulatory functions.

We here examined how the TM4SF5 C-terminus affected cellular functions differentially from other family members of TM4SF1 and TM4SF4. Using colon cancer cell lines that minimally express TM4SF1, TM4SF4, and TM4SF5, stable cell lines ectopically expressing the wild-type (WT) proteins or various deletion or chimeric mutants were developed to examine the effects on cellular functions in 2-dimensional (2D; i.e., flat) or 3-dimensional (3D) cultures. Our observations suggest that the C-terminus of TM4SF5 can replace the C-termini of the other members to further positively induce migration and invasive dissemination, although the C-termini of the three members were insignificantly distinguishable for cellular proliferation in 2D culture. The pro-migratory functions of TM4SF5 involves a structural relay from the EC2 to the C-terminus.

## RESULTS

### The C-termini of TM4SF1, TM4SF4, and TM4SF5 were relatively comparable for cell proliferation

**Figure 1:**
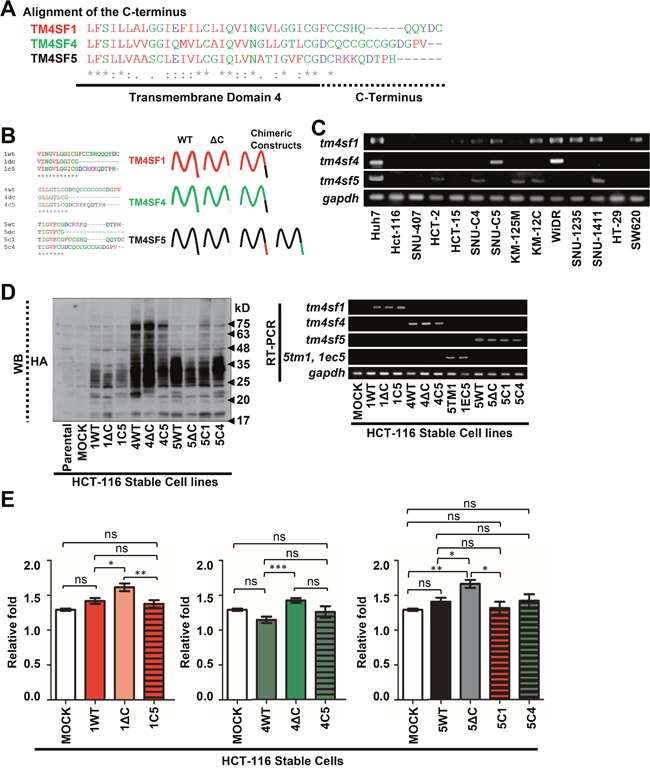
The C-termini of transmembrane 4 L six family members 1, 4, and 5 had similar effects on cell growth in 2D culture condition **A**. Alignment of the C-terminal sequences of TM4SF1, TM4SF4, and TM4SF5 showed differences in the sequences around their cytosolic tails. **B**. Schemes of the wild-type (WT) and deletion or chimeric mutants used in the study. **C**. mRNA levels of *tm4sf1*, *tm4sf4*, and *tm4sf5* in different colon cancer cells. **D**. Expression levels of the constructs (protein in left panel and mRNA in right panel) in stable HCT-116 cells. Within the TM4SF1 backbone (e.g., WT TM4SF1 [1WT], C-terminus-deleted TM4SF1 [1ΔC], and a chimera with the TM4SF5 C-terminus linked to the other parts of TM4SF1 [1C5]), the expression levels were similar each other. As for the TM4SF4 and TM4SF5 backbone, their expression levels were also comparable within each backbone. **E**. Growth of stable cells was monitored (from day 1 to day 2 after seeding) using an MTT assay, as described in the Materials and Methods. Values shown are the mean ± standard deviation (SD). *, **, and *** denote statistically significant differences at *p* <0.05, 0.005, or 0001, respectively, and ‘ns’ indicates a nonsignificant difference at *p* ≥ 0.05, by the ANOVA with Tukey's post-tests. The data shown represent three independent experiments.

Although the amino acid sequence of TM4SF5 exhibits 40-50% identity with the sequences of TM4SF1 and TM4SF4, their C-terminal sequences were found to be differ substantially (Figure 1A). To understand the significance of the respective C-termini in modulating cellular functions, mutants in which the C-terminus was deleted (i.e., ΔC) or chimeric proteins in which the C-terminus was replaced with that from another protein (i.e., 1C5 denotes the chimera in which the C-terminus of TM4SF1 was replaced with the counterpart from TM4SF5) (Figure 1B) were constructed for stable infections. We selected cell lines minimally expressing either TM4SF1, TM4SF4, or TM4SF5 for the stable introductions (Figure 1C). HCT-116 cells infected with retroviruses carrying the (HA)_3_-tagged constructs resulted in differential expression between tetraspan members, but expression levels were comparable between the different constructs sharing the same backbone (either TM4SF1, TM4SF4, or TM4SF5) (Figure 1D). During examination of the effects of each C-terminus on cell proliferation via the MTT assay, WT TM4SF1 and TM4SF5 cells exhibited increased growth, but the growth of TM4SF4-expresing cells decreased slightly, compared with control mock cells, although their ANOVA analyses indicated nonsignificant differences. All deletion mutant cells exhibited increased growth, compared with cells expressing each WT protein (Figure 1E). Interestingly, the proliferation of cells expressing the C-terminal chimeric proteins was similar to that of cells expressing each WT protein (Figure 1E).

### The C-terminus of TM4SF5 had a greater effect on spheroid growth in 3D aqueous culture than the C-terminus of TM4SF1

**Figure 2:**
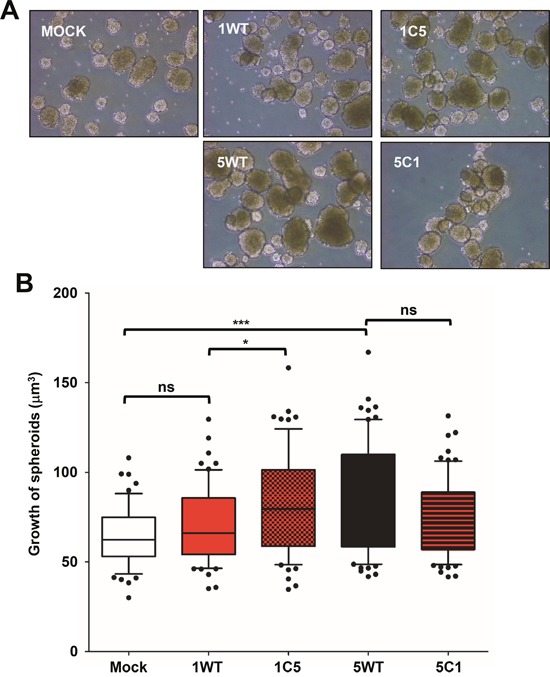
TM4SF5 and its C-terminus promoted sphere growth in 3D aqueous culture environment to a greater degree than TM4SF1 **A**. Cells (5,000 cells/condition) were cultured in Ultra-low-attachment 6-well plates for 7 days, before representative images were acquired. **B**. The diameter of spheres in the wells was determined using an Image J software, before their volume was calculated. Values shown are the mean ± SD values. *, **, and *** denote statistically significant differences at *p* <0.05, 0.005, or 0001, respectively, and ‘ns’ indicates a nonsignificant difference at *p* ≥ 0.05 by the ANOVA with Tukey's post-tests. The data shown represent three independent experiments.

We next examined how the C-terminus of each tetraspan protein affects cell growth in 3D aqueous culture. Cells were cultured on low-adhesive culture dishes, leading to the formation of spheroids (Figure 2A). The volume of spheroids formed by the stable cell lines was analyzed using Image J software. TM4SF5-positive spheroids grew significantly greater than mock control speroids, whereas TM4SF1-positive spheroids insignificantly did (Figure 2A and 2B). Interestingly, the 1C5 chimera (replacement of the C-terminus of TM4SF1 with the C-terminus of TM4SF5) promoted increased growth relative to WT TM4SF1, whereas the 5C1 chimera (i.e., the opposite chimera of 1C5) promoted insignificantly decreased growth compared with WT TM4SF5 (Figure 2A and 2B). These observations suggest that the C-terminus of TM4SF5 has a greater effect on spheroid growth in 3D aqueous culture, in contrast to the roles of the C-termini in comparably-promoting proliferation in 2D culture.

### The C-terminus of TM4SF5 could positively replace the C-termini of TM4SF1 and TM4SF4 for transwell-migration, but not *vice versa*

**Figure 3:**
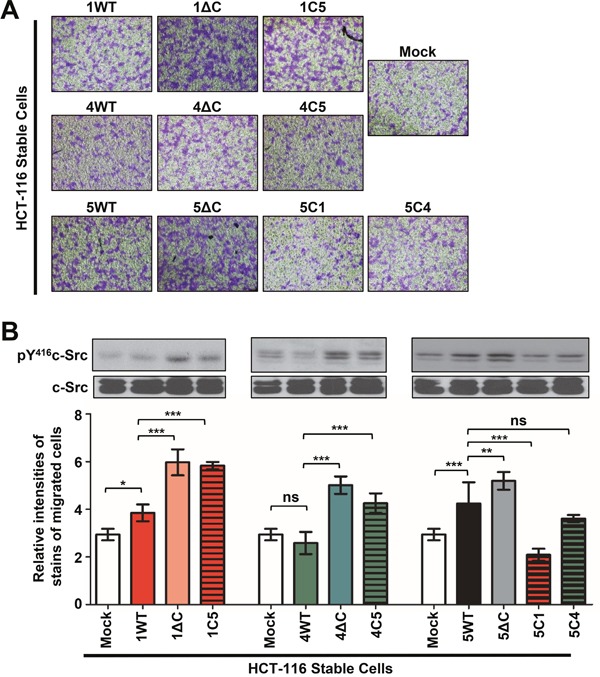
TM4SF1 and TM4SF5 but not TM4SF4 promoted enhanced transwell migration, and the TM4SF5 C-terminus could replace the pro-migratory effect of the TM4SF1 C-terminus **A**. Cells incubated with 1% BSA-DMEM for 2 h were loaded (10^5^ cells/condition) to the transwell chambers, the bottom sides of which were pre-coated with collagen I (10 μg/ml). After 10 h, cells that had migrated through the transwell chambers were stained with 5% crystal violet, and images were then acquired randomly; representative images are shown. **B**. The number of migrated cells from 10 random images per experimental condition were determined using an Image J software based on the intensity of staining. Values shown are the mean ± SD. Immunoblots for phospho-Tyr416 in c-Src and c-Src were performed using whole-cell lysates of the stable cells. *, **, and *** denote statistically significant differences at *p* <0.05, 0.005, or 0001, respectively, and ‘ns’ indicates a nonsignificant difference at *p* ≥ 0.05 by the ANOVA with Tukey's post-tests. The data shown represent three independent experiments.

We then explored how WT TM4SF1, TM4SF4, and TM4SF5 and their mutants affect cell migration. Following transwell migration, the migrated cells were stained, and the staining intensity for each condition was analyzed using Image J software. Migrations were enhanced in cells expressing TM4SF1 and TM4SF5, compared with control mock cells, whereas migration of TM4SF4-expressing cells was not significantly altered (Figures 3A and 3B). However, migration was enhanced in C-terminus-deletion mutants, compared with cells expressing the WT proteins, indicating that the C-termini play a regulatory role in transwell-migration (Figures 3A and 3B, see discussion). Furthermore, replacement of the C-terminus of TM4SF1 or TM4SF4 with the C-terminus of TM4SF5 (i.e., the 1C5 or 4C5 chimera, respectively) led to enhanced migration relative to WT TM4SF1 or TM4SF4. However, replacement of the C-terminus of TM4SF5 with the C-terminus of either TM4SF1 or TM4SF4 (i.e., 5C1 or 5C4, respectively) suppressed migration relative to WT TM4SF5 (Figures 3A and 3B). These observations also suggest that the C-terminus of TM4SF5 can positively replace the C-termini of TM4SF1 and TM4SF4 for transwell-migration, depending on influence by proper structural integrities consisting of the EC2 and/or the 4^th^ TM. In addition, the TM4SF5-mediated effect on migration was correlated with c-Src phosphorylation (Figure 3B, blots).

### The C-terminus of TM4SF5 exerted a greater effect on transwell migration than other parts of the protein

**Figure 4:**
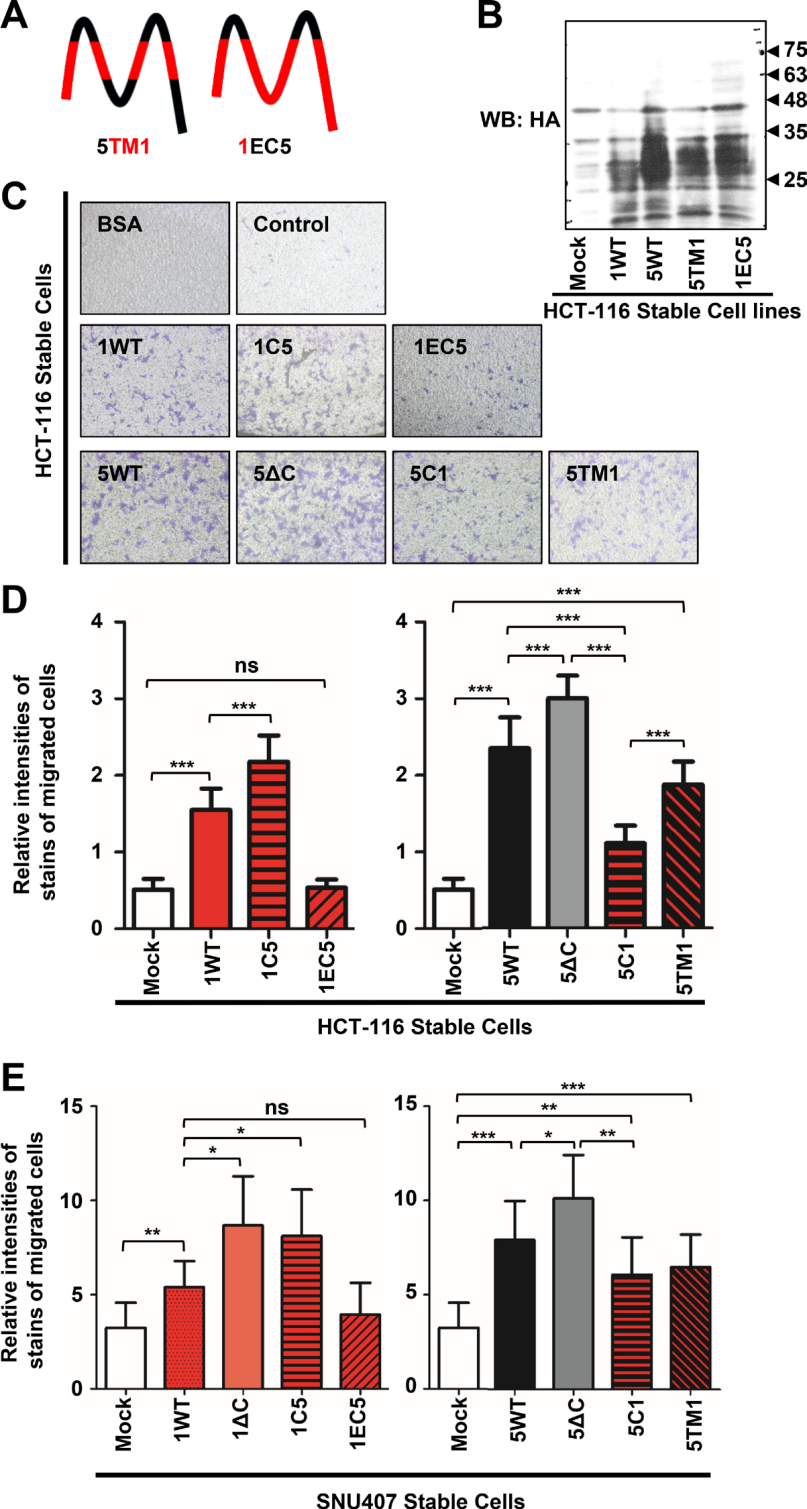
The TMs of TM4SF5, but not the EC2 alone, could promote transwell migration **A**. The schematic illustration of chimeras in which the TMs or the ECLs were exchanged between TM4SF1 and TM4SF5. **B**. Stable cells were harvested to prepare whole-cell lysates before standard Western blotting was performed using anti-HA antibody. Expression levels of the WT and chimeric mutants of TM4SF1 and TM4SF5. **C**. Transwell migration assay using transwell inserts pre-coated with collagen I (10 μg/ml) was carried out for 10 h using stable HCT-116 cells (10^5^ cells/condition), as explained in the legend for Figure 3. Images representative of 10 random images acquired per experimental condition are shown. **D** and **E**. Stable HCT-116 (D) or SNU407 (E) colon cancer cells (10^5^ cells/condition) were analyzed using transwell chambers for 13 h, as described in the legend for Figure 3. Cells that had migrated under each experimental condition were stained, and the staining intensity for 10 random images per condition was measured using Image J software. Values shown are the mean ± SD values. *, **, and *** denote statistically significant differences at *p* <0.05, 0.005, or 0001, respectively, and ‘ns’ indicates a nonsignificant difference at *p* ≥ 0.05 by the ANOVA with Tukey's post-tests. The data shown represent three independent experiments.

We next explored whether part(s) of TM4SF5 other than the C-terminus were also important for migration. We examined chimeric constructs in which the N-terminus and the TMs of TM4SF1 were conjugated with the extracellular loops (ECLs), the ICL, and the C-terminus of TM4SF5 (i.e., 5TM1) or constructs in which the ECLs of TM4SF1 were replaced with the counterparts of TM4SF5 (i.e., 1EC5) (Figure 4A). Their expressions of each of these proteins following stable infections were comparable (Figure 4B). WT TM4SF1 enhanced transwell-migration compared with control mock cells, and the chimera 1C5 also enhanced migration compared with WT TM4SF1 (Figure 4C and 4D). However, the chimera 1EC5 abolished the migration to the basal level of mock control cells (Figure 4C and 4D). Thus, it is likely that the C-terminus of TM4SF5 promotes the migration, but the ECLs alone do not when either is conjugated to the other parts of TM4SF1.

Interestingly, the TM4SF5 C-terminus-deletion mutant (5ΔC) induced markedly increased migration, compared with WT TM4SF5, suggesting that the C-terminus in the TM4SF5 context might play a negative regulatory role in migration (Figure 4C and 4D). However, the C-terminus of TM4SF5 in the TM4SF1 context (i.e., 1C5) enhanced migration compared with WT TM4SF1 (Figure 4C and 4D). Although the parts of the protein neighboring the C-terminus might be important, it is not currently clear why 1C5 and 5ΔC enhanced migration compared with WT TM4SF1 and TM4SF5, respectively. Furthermore, the 5C1 and 5TM1 chimeras did not affect migration to the levels of WT TM4SF5 or 5ΔC, indicating that the C-terminus or TMs of TM4SF1 do not promote the migration comparably to the C-terminus or the TMs of TM4SF5 (Figure 4C and 4D). The observed effects of the constructs on the migration HCT-116 cells were also observed in SNU407 colon cancer cells (Figure 4E).

### The C-terminus of TM4SF5 facilitated marked dissemination of cells from spheroids embedded in 3D collagen I gels

**Figure 5:**
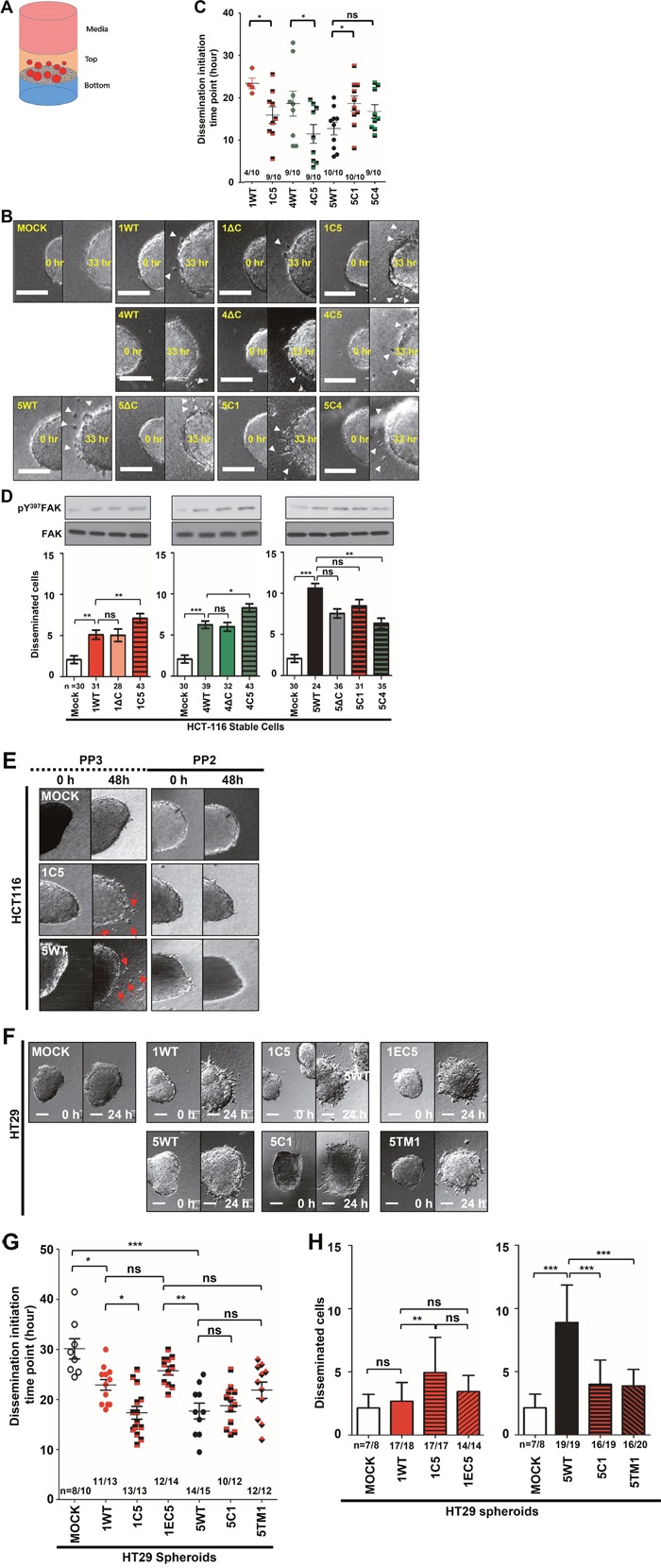
TM4SF5 and its C-terminus could positively replace the effects of TM4SF1 and TM4SF4 to promote invasive dissemination from spheroids embedded in 3D collagen I gels **A**. Scheme of the ‘on-top’ 3D ECM-surrounded culture environment, in which the cells (red) were mixed with a lower concentration (yellow-pink) of collagen I gel (0.2 mg/ml) and overlaid on the bottom dense layer with collagen I gel at a higher concentration (blue, 2 mg/ml) that was previously solidified. **B** to **E**. Representative images of the starting (each left) and ending (each right) points of the live-imaged spheroids in each condition were shown (B and E). The two-tailed unpaired Student's *t*-test was used for the analysis in (B). * denotes statistically a significant difference at *p* <0.05, but ‘ns’ insignificantly at *p* ≥0.05. The dissemination initiation times (C) or numbers of cells disseminating (D) from the spheroids were measured during the live-imaging for 36 (C) or 24 (D) h and are presented in the graph as mean ± SD values. White arrow heads depict the disseminating cells (B). Immunoblotting for phospho-Tyr397 in FAK and FAK was performed by using the whole cell lysates prepared from the spheroids in 3D collagen I gels. PP2 or PP3 (10 μM) was added to the gels during the embedding processes, before 24 h live-imaging (E). **F** to **H**. Stable HT29 cells were also processed for spheroids for live imaging of spheroids as in (B). The starting (each left) and ending (each right) points of the dissemination process during the live imaging periods were presented (F). The initiation time points during live-imaging for 24 h (G) and numbers of cells disseminating from spheroids embedded in collagen I gels (H) were presented in the graphs as the mean ± SD values. The x/y values indicate that dissemination process was observed in x cases out of y spheroids cases we examined. *, **, and *** denote statistically significant differences at *p* <0.05, 0.005, or 0001, respectively, and ‘ns’ indicates a nonsignificant difference at *p* ≥ 0.05 by the ANOVA with Tukey's post-tests. The data shown represent three independent experiments.

We next examined how invasive migration properties were regulated by the constructs using 3D collagen I gels. First, we prepared spheroids from the stable cell lines using orbital-shaking systems and selected spheroids with a diameter ranging from 70 to 100 μm, using sieves. The selected spheroids were embedded into 3D collagen I gels, with the spheroids embedded within a soft top layer (0.2 mg/ml) that was overlaid onto a dense lower layer (2 mg/ml, on-top system, Figure 5A), prior to time-lapse imaging. The period of time before the cells to disseminate from the spheroids was much shorter for WT TM4SF5-expressing cells compared with the C-terminal chimera cells (Figure 5B and 5C). Cells expressing TM4SF1 (1WT) or TM4SF4 (4WT) disseminated slower than the 1C5- or 4C5-expressing cells in which the C-terminus of TM4SF5 was conjugated to TM4SF1 or TM4SF4, respectively (Figure 5B and 5C). Interestingly, replacement of the C-terminus of TM4SF5 with the counterpart of TM4SF1 or TM4SF4 (i.e., 5C1 or 5C4, respectively) delayed the initiation of dissemination (Figure 5B and 5C). When the cells disseminated from the spheroids were counted to determine the relative dissemination rates, WT TM4SF1-, TM4SF4-, or TM4SF5-expressing cells were significantly disseminated, compared with control mock cells, and the dissemination rates of 1C5- or 4C5-expressing cells were higher than those of WT TM4SF1- or TM4SF4-expressing cells (Figure 5B and 5D). Interestingly, deletion of the C-terminus of TM4SF5 (5ΔC) resulted in a partially and insignificantly reduced dissemination rate, although the rates for cells expressing 1ΔC or 4ΔC were unaffected, compared with 1WT or 4WT cells (Figures 5B and 5D). Replacement of the C-terminus of TM4SF5 with the counterpart of TM4SF1 or TM4SF4 (i.e., 5C1 or 5C4, respectively) insignificantly or significantly, respectively, showed dissemination rates lower than TM4SF5-expressing cells (Figure 5B and 5D). Furthermore, the higher dissemination rates of 1C5-, 4C5- and 5WT (TM4SF5) -expressing cells were correlated with higher Tyr397 phosphorylation in FAK (Figure 5D, blots).

As phosphorylation of c-Src and FAK were correlated with transwell migration and/or dissemination from spheroids in 3D collagen I gels, we examined whether inhibition of c-Src using the specific inhibitor PP2 could block the dissemination of cells expressing TM4SF5- or its C-terminus-containing chimera. Whereas the negative control inhibitor PP3 did not affect dissemination, PP2 blocked the dissemination of 1C5- and WT TM4SF5-expressing spheroids (Figure 5E). In addition, the trends in the dissemination of HCT-116 cells mediated by TM4SF5 or its C-terminus were also observed in HT29 colon cancer cells stably infected with the constructs (Figures 5F, 5G, and 5H). Compared with mock control cells, both WT TM4SF1- and TM4SF5-expressing cells showed an accelerated dissemination rate (Figure 5G). Replacement of the C-terminus in TM4SF1 with the C-terminus of TM4SF5 (i.e., 1C5) or with the ECLs of TM4SF5 (i.e., 1EC5) led to differential effects. For example, compared with TM4SF1, 1C5 significantly accelerated dissemination, but 1EC5 significantly delayed dissemination (Figure 5G), suggesting that the ECLs alone did not contribute to TM4SF5-mediated dissemination, although the C-terminus of TM4SF5 positively contributed to the dissemination effect when it was conjugated to other parts of TM4SF1. Furthermore, compared with TM4SF5-expressing cells, cells expressing 5C1 or 5TM1 (a chimera in which the TMs of TM4SF5 were replaced with those of TM4SF1) showed insignificantly delayed dissemination (Figure 5G). However, the relative dissemination rate of WT TM4SF5 (5WT) spheroids was markedly higher than those of 5C1 and 5TM1 spheroids, whereas the dissemination rates of IEC5 or 1WT spheroids were insignificantly distinguishable (Figure 5H). These observations suggest that the TMs but not the ECLs alone of TM4SF5, in addition to its C-terminus, are important for regulating the dissemination rate. Collectively, these observations suggest that the intracellular parts and the TMs are important for TM4SF5-mediated c-Src activity and dissemination of cells from spheroids embedded in 3D collagen I gels.

### A structural relay (a kind of propagated conformational change) from the EC2 to the C-terminus of TM4SF5 appeared to be important for invasive dissemination

**Figure 6:**
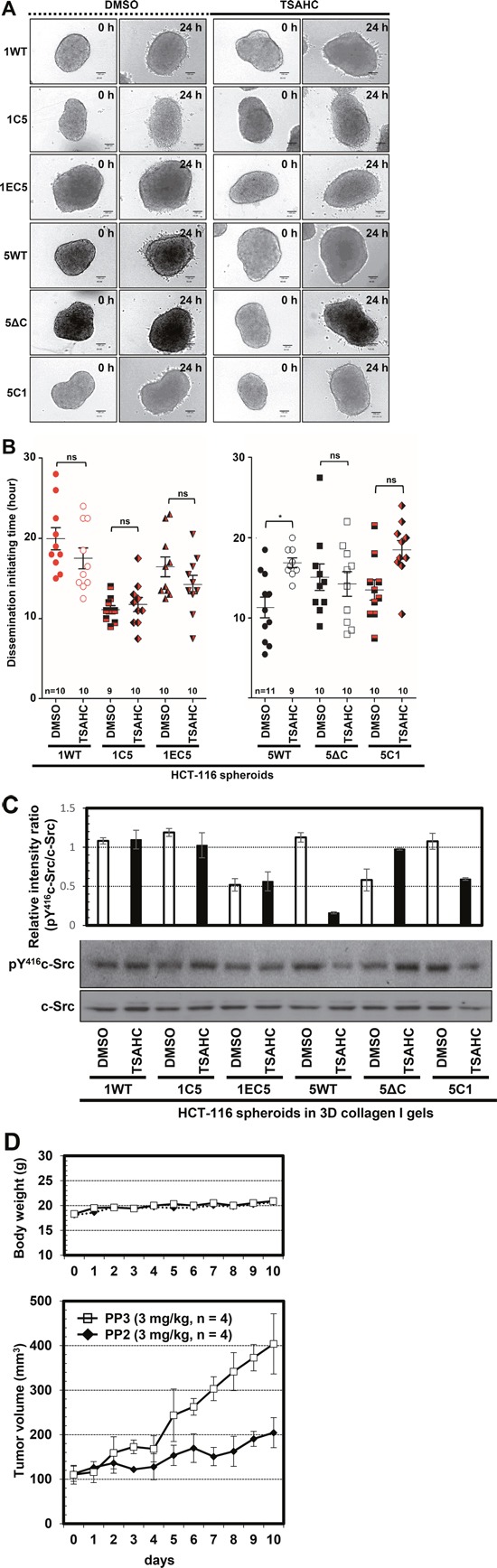
The structural relay from the EC2 to the C-terminus of TM4SF5 was important for the TM4SF5-mediated dissemination **A**. Spheroids were embedded into the 3D collagen I gels with DMSO or TSAHC (40 μM), before the 24 h live imaging. The starting (0 h) and ending (24 h) time points of imaging were shown. **B**. The dissemination initiation time of was determined. Data shown were the mean ± SD values. * denotes statistically a significant difference at *p* <0.05, and ‘ns’ indicates a nonsignificant difference at *p* ≥ 0.05 by the ANOVA with Tukey's post-tests. **C**. Whole-cell extracts were prepared from spheroids treated with DMSO or TSAHC (20 μM) for 24 h and grown in 3D collagen I environment. Relative phospho-Tyr416 levels in c-Src were determined using standard Western blotting, and the intensity of each band under each experimental conditions was measured and presented. The data shown represent three independent experiments. **D**. Hepatic SNU449T_7_ cells ectopically-expressing TM4SF5 were subcutaneously injected to mice and after the tumor volumes reached to 100 mm^3^, the mice were treated intraperitoneally with PP2 (a specific c-Src inhibitor, 3 mg/kg, n = 4) or PP3 (a negative control for PP2, 3 mg/kg, n = 4) every day for 10 days.

Cells expressing 1EC5 exhibited insignificant transwell migration and dissemination (Figures 4D, 4E, 5G, and 5H), suggesting that the ECLs alone in TM4SF5 are not critical for dissemination. However, the C-terminus and/or the TMs of TM4SF5 could be important for regulating the dissemination rate (Figure 5). We, thus, asked whether the pro-metastatic roles played by the TMs and the C-terminus of TM4SF5 required the ECLs including EC2. The anti-TM4SF5 small synthetic compound 4′-(*p*-toluenesulfonylamido)-4-hydroxychalcone (TSAHC) blocks TM4SF5-mediated migration by interrupting *N*-glycosylation or the structural integrity of the EC2 [21]. Although the reported IC_50_ of TSAHC against TM4SF5 is approximately 5 μM for 2D cell cultures and 0.3 μM for 3D aqueous spheroid culture systems (Kim and Lee, unpublished observations), we treated spheroids with 40 μM TSAHC during embedding in 3D collagen I gels. Upon treatment with TSAHC, WT TM4SF5 spheroids lost the disseminative capacity, with a significant delay in dissemination initiation, but the change in dissemination of TM4SF1 spheroids was insignificant (Figure 6A and 6B). Interestingly, 5ΔC, 1C5, 1EC5, and 5C1 spheroids maintained comparable dissemination initiation times irrespective of TSAHC treatment (Figure 6A and 6B). Therefore, cells that have both the EC2 targeted by TSAHC [21] and the C-terminus to recruit c-Src [17] were sensitive to TSAHC. Further, TSAHC treatment decreased pY^416^c-Src levels in 5WT and 5C1 spheroids embedded in 3D collagen I gels, but not in spheroids expressing other constructs, although the reduction in pY^416^c-Src level of 5C1 chimera-expressing spheroids might be less, compared with that of WT TM4SF5-expressing spheroids (Figure 6C). These observation suggests that the C-terminus of TM4SF1 linked to the TMs and the EC2 of TM4SF5 was still but less sensitive to TSAHC. Furthermore, we examined whether c-Src activity was important for the TM4SF5-mediated tumor growth by animal xenograft studies. Xenograft analysis following subcutaneous injection of hepatic SNU449T_7_ cells ectopically-expressing TM4SF5 to mice showed that the pharmacological inhibition of c-Src activity decreased TM4SF5-mediated tumorigenesis (Figure 6D).

## DISCUSSION

Because the sequence identity in the C-termini among TM4SF5 and other transmembrane 4 L six family protein TM4SF1 and TM4SF4 are very limited, compared to 40 ~ 50% identify in whole sequences, we thus examined how the TM4SF5 C-terminus affected cellular functions differentially from other family members. The results showed that TM4SF5 play greater roles, compared with TM4SF1 or TM4SF4, in promoting spheroid growth in 3D aqueous culture condition, transwell migration, and invasive dissemination from spheroids embedded in 3D collagen I gels. By contrast, all three proteins were comparable with respect to promoting growth in 2D culture environment. More specifically, the C-terminus of TM4SF5 could impart TM4SF5-mediated positive effects on cellular functions to TM4SF1 or TM4SF4 when the C-terminus of either protein was replaced with the TM4SF5 C-terminus. In addition to the C-terminus, the EC2 and the TMs of TM4SF5 appeared to be important for the TM4SF5-mediated *in vitro* and *in vivo* effects, suggesting the presence of a critical structural relay between the EC2 and the C-terminus. This relay appears to be critical for the enhancement of metastatic potential by TM4SF5.

The tetraspanins (transmembrane 4 superfamily, TM4SF) and transmembrane 4 L six family (tetraspans) proteins can form TERMs, consisting of homophilic or heterophilic protein-protein complexes with tetraspan(in)s, growth factor receptors, or integrins, resulting in the activation of unique intracellular signal transduction pathways [22, 23]. Since TM4SF5 differentially shares whole or C-terminal sequence identities with TM4SF1 and TM4SF4 [4] (Figure 1A), the sequences of the C-termini of the transmembrane 4 L six family members suggest that the proteins have unique roles in regulating cellular functions. This suggestion led us to examine the roles of the C-termini of the family members together.

The expression of either TM4SF1 or TM4SF5 is enhanced compared with normal tissues in many different types of cancer, including hepatocellular carcinoma, [6, 24]. TM4SF1 and TM4SF5 play roles in both cell proliferation leading to hepatocellular carcinoma and migration and invasion in cancer metastasis [17, 19, 24]. With respect to effects on cell function, TM4SF4 is both similar to and different from TM4SF1 and TM4SF5. In contrast to TM4SF1 and TM4SF5, which slightly increased cell proliferation in 2D cell culture condition, TM4SF4 had a slightly decreasing effect on cell growth. TM4SF4 is upregulated in injured liver in CCl_4_-treated mice [11], overexpressed in liver cancer tissues [12, 24], and is correlated with directional migration [16, 25], similar to TM4SF5 [26]. However, controversy surrounds the effects of TM4SF4 on cell growth. TM4SF4 expression is correlated with increased growth and migration via insulin-like growth factor 1 receptor activation following nuclear factor kappa B-mediated insulin like growth factor-1 induction in A549 cells [13]. Forced expression of TM4SF4 in HeLa cells causes cell density-related inhibition of proliferation, depending on the degree of its *N*-glycosylation [9]. Therefore, with respect to certain functions, TM4SF5 shows regulatory roles, similar to TM4SF4.

Although TM4SF1, TM4SF4, and TM4SF5 share a common membrane topology and regulatory roles with respect to certain cellular functions, their structural similarity is limited in the C-terminus. Interestingly, we found that the C-terminus of TM4SF5 has a greater effect on diverse cell functions, so that it can positively replace the C-terminus of other family members. It would, thus, be interesting to determine how the C-terminus of TM4SF5 plays such a critically pro-migratory role. A small-molecule anti-TM4SF5 compound TSAHC is suggested to disturb the structural aspects or *N*-glycosylation status of the EC2 in TM4SF5 [21]. Here TSAHC blocked the pro-migratory effects mediated by WT TM4SF5 but not the effects mediated by the TM4SF5 C-terminal-deletion mutant (i.e., 5ΔC), suggesting that an (allosteric) structural aspects to relay from the EC2 to the C-terminus may allow the activity of the EC2 to be transferred to the C-terminus to recruit and activate c-Src [17]. By contrast, no significant effect was observed following the application of TSAHC to WT TM4SF1-expressing cells, indicating that the EC2 of TM4SF5 but not that of TM4SF1 is responsive to TSAHC, resulting in a delay in dissemination. This confirms previous observations that TSAHC has no effects on TM4SF5-null cells [27]. To be consistent, cells expressing chimeric TM4SF1 with the TM4SF5 C-terminus (i.e., 1C5) or with the EC2 of TM4SF5 (i.e., 1EC5) did not respond to TSAHC treatment, leading to comparable dissemination initiation times and dissemination rates between DMSO- and TSAHC-treated cells. In cells expressing the chimera in which the C-terminus of TM4SF1 replaced that of TM4SF5 (i.e., 5C1), the treatment with TSAHC insignificantly retarded the dissemination initiation time and reduced the dissemination rate compared with DMSO-treated 5C1-expressing cells. This result suggests that the C-terminus of TM4SF1 might play roles similar in part or less significantly to the C-terminus of TM4SF5 based on sensitivity to TSAHC, as long as the EC2 and the TMs of TM4SF5 are available to link the structural relay to the C-terminus. Alternatively, it may not be ruled out that the TSAHC-mediated effects observed on invasion could result from changes to the pattern of TM4SF5 interactions with other cell surface partners in TM4SF5-enriched microdomains, resulting in clustering differences.

The C-terminus of TM4SF5 caused greater growth and migration in 1C5 chimera, compared with those in 1WT, whereas its deletion (i.e., 5ΔC) also increased them. Thus, it appeared that the C-terminus itself in TM4SF5 played roles in regulation of cellular behaviors in complicated manners. The C-terminus binds to inactive c-Src and active c-Src, with a preference to inactive c-Src, and TM4SF5/inactive c-Src or TM4SF5/active c-Src populations can be differentially localized along the side or leading membrane edges, respectively [17]. However, it seems that TM4SF1 and TM4SF4 cannot recruit (inactive) c-Src in major (SHE and LJW, unpublished observations). Instead, the C-terminal TM4SF1 tail is known to bind syntenin-2 to target it to TERM [19], but the binders to the TM4SF4 C-terminus are not known. However, it cannot be ruled out that TM4SF1 and TM4SF4 would also lead to c-Src activation via different signaling pathways and/or molecular sources. Therefore, the discrepancy among wildtype (5WT), deletion mutants (5ΔC) and the chimera (1C5) as for the influence on cell growth or migration might be due to differential ability of the C-terminus to bind both inactive and active c-Src, which might depend on the structural contexts and/or integrity from the EC2 to the C-terminus in TM4SF forms.

Altogether, the data of the current study showed that a structural relay from the EC2 to the C-terminus of TM4SF5 plays a significant role in promoting metastasis in 2D and/or 3D culture environments. Thus, it is reasonable to target the signaling activity of the TM4SF5 C-terminus to disrupt the effect of the structural relay from the EC2 to the C-terminus.

## MATERIALS AND METHODS

### cDNAs

Preparation of TM4SF5 cDNA was described previously [24]. TM4SF1 and TM4SF4 cDNAs were purchased from Origene (Rockville, MD, USA). Mutant DNA constructs, in which the C-terminus of TM4SF1, TM4SF4, and TM4SF5 was deleted or exchanged for one of the others, were cloned from the WT cDNAs using PCR. Chimeric 5TM1 and 1EC5 DNA constructs were described previously [19]. These DNA constructs including WT cDNAs were introduced into a pBABE-HAII-puro retroviral vector in parallel.

### Cells

pBABE-HAII-puro retroviral vectors containing each WT or mutant construct were separately cotransfected with packaging (G.P) and envelope (pDM.G) vectors into Phoenix retrovirus-producing cells. Retroviruses were harvested from the supernatant at 24 h postransfection. Polybrene was added to enhance the infection efficiency. HCT-116, HT-29 (ATCC, Manassas, VA, USA), or SNU-407 (Korean Cell Bank, Seoul National University, Seoul, Korea) cells were infected with the retrovirus and selected in 2 μg/ml puromycin for 2 days. Established stable cell lines were maintained in Dulbecco's modified Eagle's medium (DMEM) (Welgene Inc., Daegu, Korea) containing 10% fetal bovine serum (FBS) and 1% penicillin/streptomycin (GenDEPOT Inc., Barker, TX, USA) at 37°C in 5% CO_2_.

### Spheroid formation and embedding into 3D collagen type I gels

Spheroids were formed by seeding each stable cell line at 8 × 10^5^ cells in a non-adhesive petri dish in DMEM (Welgene Inc.) containing 10% FBS and 1% penicillin/streptomycin (GenDEPOT Inc.) and incubating the cells on an orbital shaker within a CO_2_ incubator (37°C and 5% CO_2_) until collection of the spheroids.

### Sphere growth assay

Cells were collected and washed twice with phosphate-buffered saline (PBS) in order to remove serum and then suspended in serum-free DMEM/F12 supplemented with 1% penicillin/streptomycin (GenDEPOT Inc.) and 2% B27 supplement (Invitrogen, Waltham, MA, USA). A total of 25 ng/ml of hEGF and hbFGF (Peprotech, Rocky Hill, NJ, USA) were added every other day. The cells were subsequently cultured for 7 days in Ultra-low-attachment 6-well plates (Corning Inc. Corning, NY, USA) at a density of 5 × 10^3^ cells/well.

### Proliferation assay

Each stable cell line was seeded in 8 wells (5 × 10^3^ cells/well) in two 96-well plates. After 24 or 48 h, cells were treated with 20 μM MTT [3-(4,5-dimethylthiazol-2-yl)-2,5-diphenyltetrazolium bromide] solution (Sigma, St. Louis, MO, USA) for 4 h at 37°C and mixed with MTT solvent for 20 min, after which the optical density was monitored at 540 nm.

### Time-lapse imaging of cells in 3D ECM gels

Time-lapse images of spheroids embedded in 3D collagen type I (2 mg/ml, PureCol^®^ from Advanced BioMatrix) gels were collected every 30 min for 24~48 h using an IX81-ZDC microscope (Olympus, Tokyo, Japan). The microscope was equipped with a 48-well Chamlide Incubator System (Live Cell Instrument, Seoul, Korea), and the environmental chamber was maintained at a constant 37°C, 5% CO_2_, and 95% humidity. Scale bars depict 100 μm. In certain cases, specific pharmacological inhibitors (PP2 or negative control PP3 at 10 μM to inhibit c-Src, or TSAHC at 40 μM to inhibit TM4SF5) were added to the gels during the embedding process.

### RT-PCR

Total RNA was extracted from cells using TRIzol reagent (Invitrogen), according to the manufacturer's protocol. Total RNA (500 ng) was reverse-transcribed using amfiRivert Platinum cDNA Synthesis master mix (GenDEPOT). The primers used for PCR are listed in Supplementary Table 1. Because the 5TM1 and 1EC5 constructs shared the same N-terminus and the EC2, these regions were used to design the PCR primers (Supplementary Table 1).

### Western blotting

Cells were grown in 6-well culture plates and harvested at 80% confluence to prepare whole-cell lysates using modified RIPA buffer containing Triton X-100 [24]. The spheroids cultured in the 3D collagen type I environment were harvested as described in a previous report [28]. Primary antibodies used for immunoblotting were as follows: phospho-Y^416^ Src, (Cell Signaling, Boston, MA, USA); c-Src (Santa Cruz Biotechnology, Santa Cruz, CA, USA); FAK, phospho-Y^397^FAK (BD Bioscience, San Jose, CA, USA); anti-HA (BioLegend, San Diego, CA, USA).

### Transwell migration assay

Stable cells were analyzed for migration using transwell Boyden chambers with an 8.0 μm of pore size (Corning, Corning, NY, USA). The membrane was coated with ECM by incubating it with 10 μg/ml collagen type I in PBS solution at 37°C for 2 h, and the control membrane was incubated with 5% bovine serum albumin (BSA) in DMEM culture medium at 37°C for 2 h. Prior to the assay, stable cells were collected in the 1% BSA-DMEM and continuously agitated for 2 h in order to nullify their adhesion signals. Cells were then seeded at 1 × 10^5^ cells per insert within 1% BSA-DMEM, and the wells were filled with the same culture medium. Migration was analyzed for 10 h (HCT-116 cells) or 13 h (SNU407 cells).

### Xenograft assay

Tumor xenograft analysis was performed, as explained previously [24]. After the tumor volume reached to 100 mm^3^, PP3 or PP2 (3 mg/kg, n = 4) was intraperitoneally injected every day for 10 days and the volumes were measured every day.

### Statistical methods

ANOVA with Tukey's post-test or two-tailed unpaired Student's *t*-test was performed to determine the significance of difference between the two groups. A *p*-value less than 0.05 was considered statistically significant.

## SUPPLEMENTARY MATERIALS FIGURES AND TABLES


