# Supplementary material for: Differential regulation of cellular functions by the C-termini of transmembrane 4 L six family proteins in 2- or 3-dimensional environment
Source: Oncotarget. 2017 Jan 25;8(8):13277–92. doi: 10.18632/oncotarget.14809 (PMC5355095; doi:10.18632/oncotarget.14809)
Supplement: Supplementary file 1 [file oncotarget-08-13277-s001.pdf]

Differential regulation of cellular functions by the C-termini of transmembrane 4 L six family proteins in 2- or 3-dimensional environment

SUPPLEMENTARY TABLE

Supplementary Table 1: The primers that used in the RT-PCR

Primer Name	Sequence
TM4SF1 Forward	GATGCTCCTGCCAGCATTTG
TM4SF1 Reverse	GCACATCGTTTGCCACAGTT
TM4SF4 Forward	TAGATGACAACGACCACCTTTC
TM4SF4 Reverse	CTCTCGGCACTTGTTCCATAA
TM4SF5 Forward	CTGCCTCGTCTGCATTGTGG
TM4SF5 Reverse	CAGAAGACACCACTGGTCGCG
1WT N-Terminus Forward	ATGTGCTATGGGAAGTGTGCA
5WT EC2 Reverse	GAGGGGGCGCCTCGCACCGAT
GAPDH Forward	TGCACCACCAACTGCTTAGC
GAPDH Reverse	GGCATGGACTGTGGTCATGAG
